# Concerning the Tenth International Medical Congress

**Published:** 1890-04

**Authors:** A. Jacobi

**Affiliations:** 110 West 34th Street, New York


### CONCERNING THE TENTH INTERNATIONAL MEDICAL CONGRESS.

110 WEST 34th STREET. NEW YORK, March 7th, 1890.

I am directed by the Secretary General of the Tenth International Congress to give the greatest possible publicity to the circular, the main points of which I herewith transmit to you with the request that they be published.

Very respectfully yours,
A. JACOBI.

